# Quantum machine learning in ophthalmology

**DOI:** 10.1038/s41433-024-03213-y

**Published:** 2024-06-29

**Authors:** Mouayad Masalkhi, Joshua Ong, Ethan Waisberg, Andrew G. Lee

**Affiliations:** 1 University College Dublin School of Medicine, Belfield, Dublin Ireland; 2 Michigan Medicine, University of Michigan, Ann Arbor, MI USA; 3 Department of Ophthalmology, University of Cambridge, Cambridge, UK; 4 Center for Space Medicine, Baylor College of Medicine, Houston, TX USA; 5 Department of Ophthalmology, Blanton Eye Institute, Houston Methodist Hospital, Houston, TX USA; 6 The Houston Methodist Research Institute, Houston Methodist Hospital, Houston, TX USA; 7 Departments of Ophthalmology, Neurology, and Neurosurgery, Weill Cornell Medicine, New York, NY USA; 8 Department of Ophthalmology, University of Texas Medical Branch, Galveston, TX USA; 9 University of Texas MD Anderson Cancer Center, Houston, TX USA; 10 Texas A&M College of Medicine, Bryan, TX USA; 11 Department of Ophthalmology, The University of Iowa Hospitals and Clinics, Iowa City, IA USA

**Keywords:** Neurological disorders, Medical research

## Introduction

Quantum computing (QC) is an advanced field of innovative technology that leverages the principles of quantum mechanics to perform computations far beyond the capabilities of classical computers [1]. At its core, QC utilizes quantum bits (qubits), which unlike classical bits can exist in multiple states (other than 0 and 1 used in traditional computing) simultaneously through superposition [1]. This allows QC to process a vast number of possibilities simultaneously [1]. Additionally, entanglement, a unique property of qubits, enables instantaneous coordination between them, which further enhances computational power [1].

The applications of QC are profound, particularly in healthcare and medicine. QC algorithms can significantly improve the efficiency of drug discovery and development by simulating molecular structures and interactions with unparalleled precision and speed [2]. Furthermore, QC has the potential to enhance diagnostic techniques and personalize treatment plans by processing and analyzing massive datasets from medical records, genetic information, and clinical trials [1].

QC may be particularly beneficial in fields such as ophthalmology, where large datasets and complex simulations are common [3]. QC has already shown promise in the simulation of eye diseases such as glaucoma [4], as well as in the discovery of new drugs [5]. The capability to process vast amounts of data, enables researchers to gain more accurate simulations of ocular conditions, which leads to better understanding and treatment strategies. This is particularly relevant in personalized medicine, where tailored treatments can be developed based on individual genetic profiles. Moreover, QC technology can be potentially valuable for managing rare ophthalmic genetic diseases (e.g., Stargardt syndrome or other forms of retinitis pigmentosa).

## Applications of quantum computing in ophthalmology

QC can significantly enhance data management from imaging techniques like optical coherence tomography (OCT), computed tomography, and magnetic resonance imaging by improving image resolution and processing speed, leading to earlier and more accurate detection of ocular, orbital, and neuro-ophthalmic diseases. Integrating QC with AI and deep learning algorithms can improve disease classification and decision-making, enhancing the diagnosis and management of diseases like diabetic retinopathy and age-related macular degeneration with greater precision [6, 7]. Furthermore, QC can enhance predictive analytics, enabling better forecasting of disease progression and outcomes, thus allowing for more proactive and tailored treatment strategies for patients, improving overall care and prognosis.

Naick et al. [8] presented the first application of QC in neural network models for classifying ophthalmological diseases using OCT images of macular drusen, choroidal neovascularization, and diabetic macular oedema [8]. Utilizing PennyLane, the model was tested on IBM’s quantum systems, integrating a hybrid quantum-classical approach with a 2-qubit QNode layer. This model was trained on 918 labelled OCT scans (414 normal and 504 abnormal) and validated on 302 scans (97 normal and 205 abnormal). The model was able to effectively categorize normal and abnormal OCT scans with an accuracy of 0.95 [8]. Future work aims to explore the broader potential of QC in disease categorization by developing a 4-class classifier with 4 qubits [8].

Topaloglu [9] explored the application of QC machine learning for the detection and classification of ocular diseases such as age-related macular degeneration, cataracts, diabetic retinopathy, glaucoma, hypertension, and pathological myopia using fundus photography. A dataset of fundus images from 1000 patients, with the images downsampled to 128 × 128 pixels to enhance processing efficiency was used as part of this study [9]. Researchers employ a quanvolutional neural network, which integrates quantum and classical convolutional layers, and quantum circuits to modify pixel values prior to neural network processing [9]. An accuracy improvement of 2.07% and a 1.979× loss function minimization compared to traditional computing methods was demonstrated [9]. The QC model was simulated using the Pennylane library on an Intel i-7 CPU, utilizing the Adam optimizer and sparse categorical cross-entropy as the loss function [9]. Overall, this shows that QC machine learning can significantly improve the accuracy and efficiency of ocular disease classification [9].

## Conclusion

Overall, QC is a groundbreaking technological advancement that uses qubits to solve complex problems beyond the capability of classical computers. In ophthalmology, QC could lead to earlier and more accurate disease detection through significantly enhancing imaging techniques. The immense capacity to perform analysis of large datasets also enables QC to develop predictive models and personalized treatment plans. By accelerating drug discovery through quantum simulations, new treatments for eye diseases can be developed more quickly and precisely. Additionally, integrating QC with AI and deep learning algorithms enhances disease classification and management and promises better outcomes for conditions such as diabetic retinopathy and macular degeneration. Large language models like OpenAI’s GPT-4 [10–12] and Google’s Bard [13, 14] As research progresses, QC methods are expected to further push the boundaries in ophthalmology.
